# Genome Editing Human Pluripotent Stem Cells to Model β-Cell Disease and Unmask Novel Genetic Modifiers

**DOI:** 10.3389/fendo.2021.682625

**Published:** 2021-06-02

**Authors:** Matthew N. George, Karla F. Leavens, Paul Gadue

**Affiliations:** ^1^ Center for Cellular and Molecular Therapeutics, The Children’s Hospital of Philadelphia, Philadelphia, PA, United States; ^2^ Department of Pediatrics, Perelman School of Medicine, University of Pennsylvania, Philadelphia, PA, United States; ^3^ Division of Endocrinology and Diabetes, The Children’s Hospital of Philadelphia, Philadelphia, PA, United States; ^4^ Department of Pathology and Laboratory Medicine, The Children’s Hospital of Philadelphia, Philadelphia, PA, United States

**Keywords:** IPS (induced pluripotent stem) cell, pluripotent stem cell (PSC), beta cell (β cell), diabetes, disease modifier, MODY (mature onset diabetes of the young), candidate gene approach, GWAS (genome-wide association study)

## Abstract

A mechanistic understanding of the genetic basis of complex diseases such as diabetes mellitus remain elusive due in large part to the activity of genetic disease modifiers that impact the penetrance and/or presentation of disease phenotypes. In the face of such complexity, rare forms of diabetes that result from single-gene mutations (monogenic diabetes) can be used to model the contribution of individual genetic factors to pancreatic β-cell dysfunction and the breakdown of glucose homeostasis. Here we review the contribution of protein coding and non-protein coding genetic disease modifiers to the pathogenesis of diabetes subtypes, as well as how recent technological advances in the generation, differentiation, and genome editing of human pluripotent stem cells (hPSC) enable the development of cell-based disease models. Finally, we describe a disease modifier discovery platform that utilizes these technologies to identify novel genetic modifiers using induced pluripotent stem cells (iPSC) derived from patients with monogenic diabetes caused by heterozygous mutations.

## Introduction

Diabetes mellitus is a worldwide healthcare problem that is rapidly increasing in prevalence. In the United States alone, over 10% of the population is affected, with approximately 1.5 million Americans newly diagnosed with diabetes each year (1). Particularly troubling is the dramatic increase in the incidence of diabetes in children, the consequences of which are expected to lead to increased complications and comorbidity as adults (2, 3). With obesity rates projected to increase in the United States over the upcoming decades, there is little chance that the trend of increasing diabetes prevalence will reverse itself (4). In addition to decreasing quality of life, diabetes is associated with significant morbidity and mortality, including retinopathy, neuropathy, cardiovascular and kidney disease (5). Diabetes also puts individuals at risk of having more complicated courses of common illnesses, with recent studies documenting increased morbidity and mortality in patients with diabetes who contracted COVID-19 (6–8).

Though often referred to as a single condition, diabetes is likely many overlapping diseases, with most stemming from pancreatic β-cell dysfunction and the disruption of glucose homeostasis due to abnormal insulin secretion and/or responsiveness (9). The two most common forms of diabetes, type I (T1D) and type II (T2D), are associated with the eventual loss of insulin-secreting pancreatic β-cells, which can occur either early (T1D) or late (T2D) in disease progression. In the case of T1D, autoimmune destruction results in β-cell death and subsequent insulinopenia, although there is increasing support for the role of β-cell stress in T1D onset (10, 11); T2D is characterized by a combination of peripheral insulin resistance and inadequate β-cell compensation, resulting in a metabolic syndrome that leads to eventual β-cell exhaustion and loss of β-cell mass (12, 13). T1D and T2D display a multifactorial etiology on both a population and individual level, likely motivated by a complex combination of genetic, epigenetic, and environmental factors (14). Furthermore, there is substantial heterogeneity within the underlying β-cell pathophysiology of each of these disorders (9).

Apart from the two major types, there are also 14 known types of monogenic diabetes, historically called MODYs (mature onset diabetes of the young), which are caused by single gene mutations that result in β-cell dysfunction (15–18). Monogenic diabetes is typically underrecognized and underdiagnosed, with identified subtypes likely making up 2-5% of all diabetes cases while additional causative genes undoubtably remain to be discovered (17). To complicate matters further, a number of MODY genes have been associated with the development of T1D and T2D (19–21), suggesting that the pathophysiology of the diabetes subtypes can often overlap. For example, there are additional genes that can cause neonatal diabetes through downstream impacts on pancreatic development, such as GATA6 and NKX2.2, which are not traditionally included as MODYs, although overlap in disease pathology occurs depending on the timing of their presentation (22). There are also numerous monogenic syndromes that have diabetes as a component in all or some affected individuals, including cystic fibrosis and Friedreich’s ataxia. While the underlying pathogenesis of monogenic and neonatal diabetes predominantly involves intrinsic defects of the β-cell, syndrome-associated forms of diabetes may result from both peripheral and β-cell defects, with the latter being understudied in many cases (15, 16). Better studies of all forms of diabetes are necessary to understanding the underlying pathophysiology of this complex disease.

### The Need for Human β-Cell Models in Diabetes Research

For decades, studies using mouse models greatly advanced our knowledge of diabetes (23). Mice are inexpensive relative to larger animal models (i.e., non-human primates), recapitulate human disease more closely than more basal organismal models, and were genetically-manipulatable even before the invention of modern genome editing techniques. As a result, mouse models continue to be incredibly valuable for the study of whole-body physiology, capable of providing complex metabolic readouts of multiorgan systems, as is the case in oral glucose tolerance tests and hyperinsulinemic euglycemic clamp studies. However, while mouse models play an essential role in the study of diabetes, important differences in rodent versus human physiology have sometimes limited the translatability of rodent datasets to the complex presentation of the human diabetes subtypes. Therefore, the development of *in vitro* human β-cell models will provide an important adjunct to *in vivo* rodent models in the study of diabetes.

While a variety of T1D and T2D mouse models exist, none have been able to comprehensively mimic human diabetes (23–25). For T1D, both genetic and chemically-induced models are used, with the latter resulting in β-cell destruction and insulinopenia, but neither method allowing for the study of the autoimmune processes that drive disease pathophysiology (24, 25). Even mouse models with an autoimmune component do not exactly resemble T1D due to interspecies differences, including well described mechanisms for the regulation of immune cell activation, homing, and target cell interactions (26). As a result, diabetes manifests differently in the two species: for example, the commonly-used non-obese diabetic (NOD) mouse strain exhibits pronounced insulitis and rapid β-cell destruction, while β-cell loss in human T1D is associated with gradual and milder infiltration of islets (27). In the case of T2D, there are a myriad of diet-induced obesity and genetic models that can be used (23, 25). However, the complex polygenic nature of human obesity can be difficult to model using inbred mice strains, and observed effects of sex, age, and epigenetic factors on diet-induced obesity may not be the same between species, though these differences may provide some insight into genetic loci that result in phenotypic variation (23).

While mouse models have yielded significant insights into monogenic causes of diabetes, they do not always fully recapitulate the human disease. One example involves GATA6 and GATA4, members of the GATA family of transcription factors, which are the most highly expressed isoforms in the pancreas. Heterozygous, largely *de novo*, mutations in GATA6 are the most common cause of pancreatic agenesis, resulting in neonatal diabetes as described by multiple groups (28–37). Though less common, GATA4 haploinsufficiency can also result in neonatal and early childhood-onset diabetes (38, 39). However, in mice, GATA4 and GATA6 appear to be completely redundant isoforms, as the loss of a single allele of either *GATA4* or *GATA6* does not appear to impact pancreatic development or glucose homeostasis, and the loss of three of the four Gata4/6 alleles is needed to recapitulate the human phenotype (40, 41). Another example is the most common form of monogenic diabetes, MODY3, caused by heterozygous mutations in the transcription factor HNF1α. Mice with heterozygous mutations in *HNF1A* are healthy (42) and mice with *HNF1A* null mutations can have a diabetic phenotype, but with significant variability dependent on genetic background (43). These results suggest that there are complex, human-specific genome-phenotype interactions that must be additionally investigated using human models. Therefore, the combined and complementary use of both *in vitro* human β-cell models and *in vivo* rodent models will greatly advance our knowledge of the pathophysiology of diabetes.

### The Emergence of the Stem Cell-Derived β-Cell as a Model

While mouse models have and certainly will continue to advance our knowledge of diabetes, β-cell-centric research in diabetes has unfortunately been hindered by the lack of human models. Most immortalized β-cell lines are rodent-derived, though several human lines, including the EndoC-βH1 (and subsequent βH2 and βH3) line, are becoming more widely used (44, 45). However, β-cell lines can exhibit differences from *in vivo* human β-cells due to their immortalized status, can be difficult to genetically manipulate, and cannot be used to study β-cell development. While the use of cadaveric human islets in research has greatly expanded due to the success of programs such as the Network for Pancreatic Organ Donors with Diabetes (https://www.jdrfnpod.org) and the Integrated Islet Distribution Program (https://iidp.coh.org), these resources unfortunately remain scarce and precocious. Furthermore, the genetic, epigenetic, and environmental characteristics of donors are largely unknown, while islets themselves cannot be genetically manipulated efficiently.

To address these limitations, great strides have been made over the last two decades in the development of human pluripotent stem cells (hPSC), a term which includes both embryonic stem cells (ESC) and induced pluripotent stem cells (iPSC) [reviewed in (46, 47)]. Efficient methods for the production of hPSCs have completely changed the face of biomedical research and have opened new avenues of therapeutic development for a multitude of diseases. The subsequent development of techniques to differentiate hPSCs into pancreatic β-cells have enormous potential to contribute to the study of diabetes. Built upon foundational research within mouse developmental biology [reviewed in (48)], modern techniques of stem cell differentiation leverage known inductive signals that drive development *in vivo* by replicating these signals temporally and spatially to drive development *in vitro* [reviewed in (49)]. The first lab-guided hPSC differentiation protocols were developed to generate definitive endoderm (50), followed quickly by protocols capable of driving hPSCs towards pancreatic progenitors and endocrine cells (51). While these protocols initially required that pancreatic progenitors be transplanted into mice to mature into functional β-cells, current protocols can achieve functionality *in vitro* without transplantation (52–54) This field has become robust with technical advancements being published regularly by laboratories around the world, resulting in the generation of β-like cells that are closer and closer to their natural counterparts, though continued optimization of functionality is required (55–62).

With advances in directed *in vitro* differentiation, stem cell-derived β-cells provide a tremendous opportunity to study pancreatic development and endocrine diseases in a human model system, particularly when combined with recent advances in genome editing technology. Using clustered regularly interspaced short palindromic repeats (CRISPR)-Cas9 technology (63, 64) (see *Section 2*), targeted mutations in hESC lines can be made, generating mutant and control isogenic lines that avoid confounding results due to differing genetic backgrounds. In addition, iPSCs can be generated from reprogramed patient donor blood or skin fibroblasts (46, 47), resulting in a unique platform within which to study the specific contribution of single mutations to β-cell development and/or function. While these systems certainly have caveats, including expense, labor-intensiveness, and lab-to-lab variability, the expanding use of stem cell-derived β-cells stands to drive our knowledge of diabetes pathophysiology forward beyond the prior limitations of mouse and cell line models. In this review, we will review the current methods of genome editing in hPSCs, discuss how this can be applied to the evaluation of candidate genes in the study of diabetes, and examine the use of stem cell-derived β-cells as a platform for the identification of novel genetic modifiers of diabetes.

## Genome Editing in hPSCs

The development of genome editing technologies capable of selectively targeting sites within the human genome has revolutionized our ability to investigate the genetic underpinnings of disease. In the case of diabetes, ESCs and patient-derived iPSCs from multiple genetic backgrounds can now be genetically edited and paired with lab-guided differentiation protocols to build powerful and scalable cell-based models of multiple diabetes subtypes (65, 66). Genome modifications in each system are achieved through nuclease localization with a target sequence, the induction of a double stranded DNA break (DSB), and the activation of endogenous cellular DNA repair mechanisms such as homologous recombination (HR) and non-homologous end-joining (NHEJ) (67, 68). Several types of gene modification can be accomplished through these mechanisms, including (1) ‘gene disruption’ through the addition/subtraction of short nucleotide sequences and frame shift mutation induction (2), ‘gene correction’ through targeted base substitutions that restore gene function using a homologous donor DNA construct as a template, and (3) ‘gene addition’ through the introduction of a complete transgene into a specific locus. Here we briefly review several of the most popular methods for the selective editing of hPSCs, each of which exhibit advantages and disadvantages when editing specific cell types (69).

### Zinc Finger Nuclease (ZFN) and Transcription Activator-Like Effector Nuclease (TALEN)

Zinc finger nuclease (ZFN) and transcription activator-like effector nuclease (TALEN) are structurally similar in that they both rely on the C-terminal *Fok*I endonuclease domain to generate DSBs within a targeted sequence (70–72). ZFN architecture combines multiple zinc finger protein DNA-binding domains (motifs) (73) with the nuclease domain of the *Fok*I restriction enzyme that performs optimally when targeting long (12-18 bp) and unique sequences within the eukaryotic genome (74). In contrast, TALENs employ multiple transcription activator-like effector (TALE) DNA binding domains – a class of proteins isolated from the *Xanthomonas* bacteria that have evolved to alter the transcription of host plants (75). In either case, the two distinct regions of the nuclease each perform a unique function, with zinc finger motifs or TALEs binding to DNA while the *Fok*I nuclease domain induces a DSB within a target sequence upon dimerization (76, 77).

While structurally similar, there are distinct advantages and disadvantages of each system. Typically, ZFN-based platforms afford greater flexibility in targeting, while also allowing for independent optimization of the two subunits for simplified retargeting (78). Drawbacks of ZFNs include the cost of application, a suite of complex design constraints that must account for context-dependent interactions between fingers (79), and a higher prevalence off-target effects and translocations than other methods (80). In contrast, the highly conserved stretches of 33-35 amino acids (AA) that TALEN-based approaches employ addresses many of the design complexity concerns of ZFNs, while maintaining high cleavage activity rates, a broad targeting range specificity, and improved cytotoxicity (81). However, TALEN-based approaches have been shown to produce off-target effects and suffer from dramatically lower efficiencies when targeting sequences that are methylated or do not include thymidine (82, 83). Comprehensive reviews that provide specific recommendations for the design and reproducible integration of ZFN (78, 84) and TALEN-based (85) genome editing approaching in hESCs and iPSCs are available elsewhere.

### Clustered Regularly Interspaced-Short Palindromic Repeats (CRISPR)/Cas

(CRISPR)/Cas-based gene editing platforms have become incredibly popular tools to modify the genome of hPSCs since the introduction of the technology in 2012 (86). Based on the adaptive immune system of bacteria and archaea, CRISPR was made possible by the discovery of DNA fragments within the *E. coli* genome from past viral and bacteriophage invaders known as clustered regularly interspaced short palindromic repeats (CRISPRs). Unlike ZFNs and TALENs which use proteins, CRISPR loci are transcribed during viral infections to produce an RNA-guided DNA endonuclease that selectively binds and cuts invading viral DNA (87). CRISPR sequences exhibit a repetitive pattern, wherein short DNA sequences (24-48 bp) are followed by their reverse complement and a protospacer that matches part of the viral genome. Through coordination with RNase III, CRISPR-associated (Cas) proteins, and trans-activating CRISPR RNA (tracrRNA), long RNA transcribed from CRISPR loci are cleaved into short, spacer-derived RNA (crRNA) (88). TracrRNA and crRNA then act together to guide the Cas9 protein to a target cut site located within the genome of an invading virus, causing a DSB [for a review, see (89)].

When used in genome-editing platforms, tracrRNA and crRNA can now be combined into a single “guide RNA” (gRNA) molecule (86) and administered with the Cas9 protein to selectively cut target DNA sequences (90). Multiple CRISPR/Cas9 systems have been developed specifically for hPSCs that are capable of editing or replacing genome sequences (91, 92) and are quickly replacing TALEN-based systems due to their ease of generation, efficiency, and cytocompatibility (93, 94). Drawbacks of CRISPR-based methods include the re-cutting of target regions after DSB repair and the prevalence of erroneous insertion or deletions (indels) on the non-targeted allele, making the generation of single allele edits difficult. Recent protocols, including one from our laboratory (95), address these issues through the use of two single stranded oligonucleotide repair templates, with one expressing the desired sequence change and the other maintaining the normal sequence. These repair templates also contain silent mutations that prevent gRNA recognition and re-cutting, facilitating the selective editing of a single allele with an average efficiency of close to 10%. In addition, off target cutting of CRISPR/Cas9 at other sites in the genome is also an issue but it can be mitigated. First, in the hPSC system, off target cutting is less prevalent than in somatic cells, mostly likely due to the fact the pluripotent stem cells are very sensitive to DNA damage and cells that have undergone multiple DNA cuts are less likely to survive (96). Second, careful design and testing of potential off target sites can also be used to minimize the impact of off target cutting. Overall, genome editing technologies in hPSCs are advancing to the stage where virtually all coding mutations can be repaired or introduced in a single allele manner, an important advance given the majority of monogenic genetic diseases of the pancreas are caused by heterozygous mutations.

## The Role of Candidate Gene and GWAS Approaches in the Study of Diabetes

The availability of standardized laboratory protocols for the generation of glucose responsive β-cells from hPSCs that have undergone selective genome editing has the potential to dramatically increase our knowledge of genes that contribute to diabetes. Traditionally, the functional contribution of genes to disease states has come from the deletion or mutation of a single gene target. The use of candidate genes, chosen as they are known clinically to cause disease, has been employed for the study of monogenic diabetes. However, given the increasing ease of genome and exome sequencing, comparisons of genetic variants between diabetic and non-diabetic populations through GWAS analysis using genome sequencing has generated a large list of variants associated with all forms of diabetes, most of which are in non-coding regions of the genome (97–100). Newly developed stem cell platforms can be used to target these variants, initially by targeted mutation of the gene thought to be regulated by a given variant (101). The direct targeting of a non-coding variant has been studied in neonatal diabetes caused by GATA6 (102), and similar approaches could also be used for variants associated with more common causes of diabetes. Care will need to be taken as it is possible that most non-coding variants may have a small impact on gene expression and disease penetrance on their own. While we are still in the early stages of utilizing these approaches in stem cell-derived β-cells to interrogate the roles of specific genes in β-cell development and function, we predict that this will become more commonplace and contribute to our understanding of β-cell physiology and disease.

### Studying Known Causes of Monogenic Diabetes

There are dozens of types of diabetes caused by single gene mutations, including monogenic, neonatal and syndrome-associated diabetes (15–17, 103–105). Monogenic forms of diabetes are often caused by heterozygous coding mutations within genes that influence β-cell function [reviewed in (106)]. Many forms of monogenic diabetes have been recognized for decades and more gene causes are likely to be found in the upcoming years with the increasing use of exome and genome sequencing (17, 18). In addition, many of the genes associated with monogenic diabetes have numerous reported mutations with potentially different consequences on protein function and therefore on β-cell dysfunction (107–109). Stem cell-derived β-cells may provide a platform in which to investigate some of these polymorphisms, and may help to provide some insight into genotype-phenotype correlations.

The modeling of monogenic diabetes using stem cell-derived β-cells has been extensively described. To date, hPSC lines have been made with mutations in HNF1A (110–114), HNF1B (112, 115, 116), HNF4A (112, 117–119), PDX1 (107, 120, 121), KCNJ11 (122), GCK (112), and CEL (112). Of the studies listed, only two used genome-editing to make mutant hESC lines (113, 121), with the remainder generating patient-derived iPSC lines. While some studies simply described the derivation of iPSC lines from patient samples, most publications included lab-guided differentiations to β-cells and subsequent studies on β-cell gene expression and biology (111, 113–115, 117–119, 121).

MODY3, caused by heterozygous mutations in the transcription factor HNF1α, is the most common form of monogenic diabetes (15, 103) and is currently the most extensively-researched monogenic form of diabetes using stem cell-derived β-cells. HNF1α has been of particular interest because of its additional association with T1D and T2D in several large population studies (19–21, 123). Several studies on the role of HNF1A has been performed in mice, but mouse models with heterozygous deletion of *HNF1A* do not have diabetes and thus have provided limited insight into MODY3 (124). To date, three publications from different groups have modeled HNF1A-deficiency in stem cell derived β-cells, with two employing patient-derived iPSC lines (111, 114) and the other using hESC lines (113). These studies have yielded significant insights into the complex role HNF1α plays in controlling β-cell development, metabolism and function and have discovered new downstream targets of this transcription factor that had not previously been identified in mouse studies.

While the underlying pathogenesis of monogenic diabetes results from intrinsic β-cell defects, the role of β-cell dysfunction in many syndrome-associated forms of diabetes, such as cystic fibrosis (CF)-related diabetes, is largely unknown (105). There is significant interest in these fields to generate syndrome-related iPSC lines for use in multiple areas, but this will ultimately aid in the study of rare causes of diabetes by providing accessible resource lines. Groups have already generated stem cell derived β-cells to model β-cell dysfunction in Wolfram syndrome (125, 126) and Friedreich’s ataxia (127). In addition, CF iPSC lines have been made and differentiated in the pancreatic ductal endothelium to study the effects of CF-related pancreatic exocrine function (128). These lines and others generated for non-diabetes study can always be used to produce stem cell derived β-cells and advance our knowledge of these understudied forms of diabetes.

Another avenue of study using stem cell derived β-cells as a model is to focus on genes that are thought to play a role in β-cell development, identity, or function but that may not have been described as monogenic causes of disease. In an impressive paper by Zhu and colleagues, researchers used genome editing to generate hPSCs knockout lines to further probe the role of 8 known pancreatic transcription factors, including PDX1, RFX6, PTF1A, GLIS3, MNX1, NGN3, HES1 and ARX (121). Many of these factors had only been studied previously in rodent models and, through lab-directed differentiation, their role in human β-cell development and function could be interrogated for the first time. This reverse candidate approach using stem cell derived β-cells will provide a significant basis for future advances.

As genome editing techniques improve and become more well-established, the field is turning more to the use of isogenic lines in which to study the contribution of a specific genes on β-cell physiology. This involves making targeted mutations in hESC lines or correcting mutations in patient-derived iPSC lines, generating mutant and control isogenic lines that avoid confounding results due to differing genetic backgrounds. In all studies above using patient-derived iPSC lines to study monogenic diabetes, the mutant stem cell-derived β-cells were compared to unaffected family members or unrelated wild type iPSC lines and not isogenic controls (111, 114, 115, 117–119). With the increasing use of CRISPR/Cas9 technology, we advocate the use of at least 2 pairs of isogenic lines, examining a single clone for each, for interrogating the impact of a given genome alteration. Alternatively, if using a single stem cell line, the examination of several genome edited clones has been suggested by leading stem cell journals such as *Stem Cell Reports* (Information for authors). We would argue that 2 isogenic pairs is superior because it controls for both artifact due to an acquired mutation in a single genome edited clone as well as confirm any phenotype is general enough to be seen in 2 independent genetic backgrounds. The use of a single edited clone per isogenic pair we feel is a good tradeoff between the effort required to differentiate and analyze these clones while still minimizing the impacts of clonal artifacts.

### Leveraging GWAS to Identify and Validate Candidate Genes in Diabetes

The cause of T1D and T2D is likely a complex combination of genetic, epigenetic and environmental factors (14). In addition, there is substantial heterogeneity within each of these disorders, so that the disease-causing combination in each affected individual is slightly different (9). Therefore, a single gene-to-disease strategy is not always effective in the study of T1D and T2D. Technological advancements in next generation sequencing, combined with the targeted efforts of several consortia, continue to expand the size and scope of available genomic datasets from diabetic patients (129–131). Previously, the identification of diabetes-linked genes was the product of quantitative trait mapping (QTL), obtained through the cross of genetically engineered mice (132–134). Over the past decade as the cost and availability of sequencing technology has improved, GWAS have identified more than 60 loci for T1D (135) and more than 240 associated with T2D (136), with the hereditability of each now explaining approximately 15% (137) and 25-80% (138) of the disease-risk for each subtype, respectively.

The explosion of available GWAS datasets for both T1D and T2D can be leveraged by using stem cell derived β-cells. Using this technique, novel genes that are revealed by GWAS, individual exome, or genome sequencing associated with diabetic populations can be targeted for study either in isolation or as part of co-cultures where interactions between adipocytes, immune cells, critical biological components and β-cells are replicated *in vitro* (139–141). Through the use of stem cell derived β-cells, these systems can ascertain whether a specific locus causes β-cell-intrinsic dysfunction, while also probing the contributions of the surrounding environment. For example, polymorphisms in the human leukocyte antigen (HLA) DR and DQ alleles increase T1D-risk by altering T-cell binding [reviewed in (142)], which is predictably a β-cell-extrinsic effect that can be observed in cell culture studies. Alternatively, some polymorphisms in the insulin (INS) gene, a β-cell-specific gene, have been described to influence T1D risk due to changing insulin mRNA production in the thymus altering immune tolerance (143, 144). However, other mutations in INS lead to neonatal diabetes, thought to be caused by β-cell death due to increased cell stress from misfolded insulin protein [reviewed in (145)]. While the difference between polymorphisms and mutations may be determined by their prevalence in the population, modeling these gene differences in stem cell derived β-cells may prove useful for understanding their significance.

While GWAS studies can yield a potential target gene which could be directly involved in disease, sometimes they identify an associated region of unclear significance. GWAS comparing the islets of diabetic and non-diabetic individuals suggest that most T2D-associated variants do not reside in coding regions (146, 147). In order to understand the role of these variants, iPSC banks from T1D, T2D and non-diabetic patients can be used to probe these differences on a multigene scale. Multiple iPSCs from T1D and T2D patients have been made (148–151), and there are consortia and foundations that are focused on making larger banks of available diabetes and non-diabetes iPSC lines, including the Human Islet Research Network (HIRN, https://hirnetwork.org/hpap-overview) and the New York Stem Cell Foundation (NYSCF, https://nyscf.org/research-institute/repository-stem-cell-search/). Several groups have also recently performed lab-directed differentiations on patient-derived iPSC lines to generate stem cell derived β-cells to examine broad molecular and functional differences (150, 151). Unlike the need for the generation of isogenic lines in the study of monogenic diabetes, making banks of T1D, T2D, and non-diabetic stem cell derived β-cells can be used to study many factors contributing to β-cell pathophysiology in diabetes. One caveat of the use of non-isogeneic lines is that differences in genetic background amongst disease and control lines leads to tremendous variability in phenotype and necessitates large sample numbers to dissect the underlying biology.

## Genetic Disease Modifiers and Diabetes

The way genetic factors interact with disease can be highly variable. Even in canonical examples of monogenic Mendelian diseases such as cystic fibrosis and sickle-cell anemia where a disease endophenotype is linked to a single mutation (152), fraternal twins that reside within the same household can present vastly discordant phenotypes (153). The results of longitudinal twin studies add to a growing body of clinical evidence that underscores the importance of ‘disease modifiers’ that alter the penetrance, expressivity, rate of progression, and/or presence of disease endophenotypes through the modification of disease-linked genes (154, 155). While the terminology used to describe the mechanisms of oligogenic inheritance continues to evolve, for the purposes of this review we have chosen to classify genetic disease modifiers within two groups based on their mode of action, or as either ‘protein coding’ or ‘non-protein coding’.

Protein coding disease modifiers typically affect the phenotypic expression of a disease through mutations in the coding sequence of intact genes, leading to changes in protein function (156). These changes can be either sufficient to elicit a diseased state (i.e., a frame shift mutation within a coding sequence of an important functional protein), or can affect the molecular expression of other disease-linked genes through alterations in regulatory DNA such as promoters and enhancers (e.g. modifier genes) (157). In contrast, non-protein coding disease modifiers include non-coding regulatory elements and non-coding RNAs (ncRNA) (158). In either case, disease modifiers can act to either enhance, silence, or modify the expression of genes that can modify the activity of important proteins whose dysregulation result in changes in the penetrance, expressivity, and/or presence of a disease endophenotype.

Diabetes is a complex disease wherein patients express significant heterogeneity in the progression, clinical phenotype, and response to treatment. T1D and T2D show clear evidence of a genetic component and familial reoccurrence (159, 160), with observed associations with lifestyle, obesity, and cancer playing a particularly significant role in T2D (161). In the face of this variability, it is important to note that the direct influence of specific mutations within protein-coding regions on the etiology of diabetes have been described (162, 163). However, while allelic variants have been shown to confer an increased risk of T1D/T2D, other subtypes of diabetes, such as monogenic diabetes, are causally linked to single mutations, as described above. Apart from changes in coding sequences, there is substantial evidence that disease progression and severity of all forms of diabetes results from the interaction of multiple non-coding genetic, epigenetic, and environmental factors, which act in concert to cause β-cell dysregulation and islet dysfunction (164, 165).

The influence of protein coding and non-protein coding disease modifiers on each of the diabetes subtypes remain active areas of research. In the case of monogenic diabetes, modifier genes have been shown to explain some degree of clinical variability (166), while several studies suggest that non-coding disease modifiers influence the development of gestational diabetes (167, 168). In this section, we provide a brief overview of the two classes of disease modifiers, discussing known associations with the diabetes subtypes when available. To facilitate the further discovery of such mechanisms, we then outline a disease modifier discovery platform that leverages recent advancements in RNA sequencing, genome editing, and laboratory-guided stem cell differentiation to identify genetic disease modifiers of genetic disease caused by heterozygous mutations, using monogenic diabetes as a model. Given the limited availability of research on this topic, it is our hope that the platform outlined here will support the discovery of novel protein coding and non-protein coding disease modifiers that can help explain the heterogeneity observed in diabetes subtypes.

### Protein Coding Disease Modifiers of Diabetes

The influence of modifier genes and allelic heterogeneity on human disorders has been the subject of an ongoing discussion within medicine for over a century (169), with multiple parallel avenues of investigation within genetics (e.g. epistasis, oligogenic inheritance) dedicated to understanding the effect of one gene/allele on the phenotypic expression of a second gene/allele (154, 155). In the case of diabetes, there is a growing body of evidence that some subtypes may be the result of oligogenic inheritance, wherein the underlying etiology of the disorder is primarily genetic, but still requires the synergistic action of several genetic modifiers at disparate disease-linked loci (156, 170, 171). In this continuum between classical Mendelian and complex traits, possible protein coding disease modifiers include allelic heterogeneity that results from mutations within disease-linked loci, the activity of modifier genes that regulate others with important roles in glucose homeostasis, and the presence/absence of single nucleotide polymorphisms (SNPs) that are either necessary or sufficient to change the presence, penetrance, expressivity/heritability, or rate of progression of a disease.

As GWAS datasets expand to include sampling of diabetic patients from varied ethnic backgrounds that present different endophenotypes, the technology is poised to assist in the identification of candidate disease-linked genes and SNPs that reside within ‘modifier loci’ (172). While hundreds of candidate genes that are linked with increased diabetes-risk have been identified, the mechanisms underlying their action often remain unclear. One of the first identified and best understood examples of how genetic protein coding disease modifiers modulate the phenotypic expression of diabetes are the multiple identified polymorphisms within the base pair sequence of the HLA region of chromosome 6p21.3 on T1D (173). In this case, variation within the coding sequence of the HLA DR and DQ alleles produce changes in the amino acid sequence of cell surface receptors, altering their binding affinity to T-cells and increasing T1D-risk [reviewed in (142)].

Apart from polymorphisms in the HLA region, coding mutations within genes that encode important pancreatic transcription factors (TFs) have also been shown to modify the phenotypic expression of diabetes (162, 163). Coding mutations within the TCF7L2 (174), PDX1 (107), HNF1A (108), HNF4A (175), and TM6SF2 (176) genes can result in the dysregulation of blood glucose homeostasis by altering TF expression or imparting direct functional consequences on β-cell or islet function through alterations in a TF’s amino acid sequence. For example, hundreds of missense mutations within PDX1 coding regions have been identified, with mutations within the transactivation domain reducing gene activation and impairing both β-cell development and function (107). In the case of HNF1A, 11 rare coding variants have been identified that result in a >40% reduction in transcription and are strongly associated with monogenic diabetes (MODY3) in the general population (108, 109). Similarly, a number of coding SNPs can impart T1D and T2D susceptibility within groups with a shared ancestral heritage, including SNPs in the SUMO4 (177) and MGEA5 (178) genes, identified within Japanese and Mexican populations, respectively.

### Non-Protein Coding Disease Modifiers of Diabetes

Recent advances in targeted RNA sequencing technology (RNA Seq, RNA CaptureSeq) have greatly expanded our understanding of transcriptomics (179), underscoring the potential importance of regulatory elements in the control of disease-linked genes (180–182). Rather than coding for a protein directly, non-coding RNA (ncRNA) regulate the transcriptional or post-transcriptional production and modification of proteins. ncRNA make up 98% of the transcripts in the human genome, can be either trans- or cis-acting, and are classified into groups according to their length, morphology, and function (179, 183). ‘Short’ ncRNAs are less than 200 bp in length and perform a diversity of functions during gene regulation, protein synthesis, and the post-translational modification of proteins. Short ncRNA include nuclear RNAs (snRNAs), small nucleolar RNAs (snoRNAs), micro-RNAs (miRNAs), and transfer RNA (tRNA), to name a only a few (184). In contrast, long non-coding RNAs (lncRNAs) are 200 bps or longer and are generally only involved in the regulation of gene transcription and epigenetic regulation, although in some rare occasions they may produce peptides (185).

To date, the influence of non-protein coding disease modifiers on the pathogenesis of diabetes remains underdefined, providing an exciting avenue for future research. GWAS comparing the islets of diabetic and non-diabetic individuals suggest that most T2D-associated variants do not reside in coding regions (146, 147), adding to a growing, yet sparse, body of evidence that glucose homeostasis is heavily controlled by the activity of non-coding regulatory elements (186). For example, thousands of miRNAs and lncRNAs have been isolated from islets (136), with preliminary evidence suggesting that some miRNA are required for islet development in mice (187) and β-cell function (188, 189). LncRNA in particular have been linked to several important processes in diabetes (190), with overexpression resulting in enhanced cell proliferation and fibrosis in the early state of diabetic nephropathy [LncRNA CYP4B1-PS1-001 (191)].

Non-coding single nucleotide polymorphisms (ncSNPs), or variations in a single DNA base pair that code for non-coding regulatory elements, can also act as disease modifiers of diabetes (182). More than 90% of disease-associated SNPs are located within non-coding regions, resulting in possible functional variants of promoters, enhancers, and ncRNA genes (192). Through this mechanism, ncSNPs within important regulatory regions can alter the splicing, binding, degradation, or sequence of a ncRNA, which in turn can modulate the activity of multiple regulatory elements that act to control other cellular processes, such as transcription factor binding and chromatin states (193, 194). As an example, a recent study from our laboratory that used genome editing to knock-out *HNF1A* in hESCs identified a human-specific lncRNA (*LINKA*) that is a target of HNF1A and is necessary for normal mitochondrial respiration within stem cell-derived β-cells (113). Given that there is recent evidence that islet-specific lncRNA and transcription factors co-regulate genes associated with enhancer clusters (195, 196), we expect that additional studies that expand upon the functional consequences on ncSNPs and the potential targets of lncRNA in human islets have great potential to explain some of the clinical heterogeneity each diabetes subtype (197, 198).

### Patient iPSCs as a Diabetes Disease Modifier Discovery Platform for Monogenic Diabetes

The discovery of genetic modifiers of diabetes have been slowed by the complex presentation of the diabetes subtypes, with the cause of each existing on a multi-dimensional continuum of genetic, epigenetic, and environmental factors (14). However, while progress has been hindered in some areas, success has been achieved over the past two decades within monogenic diabetes, with advancements in molecular genetics enabling the definition of discrete etiological disease subtypes that can inform preventative treatments through precision medicine (199). As discussed in section 3, monogenic forms of diabetes result from coding mutations in single identified genes which cause β-cell-intrinsic dysfunction. This has allowed for targeted studies focused on elucidating the role of the specific causative gene in β-cell development and function. However, disease penetrance and presentation can vary among individuals with the same underlying pathogenic mutation, suggesting that additional factors can influence the genotype-phenotype association (15–18).

**Figure 1:**
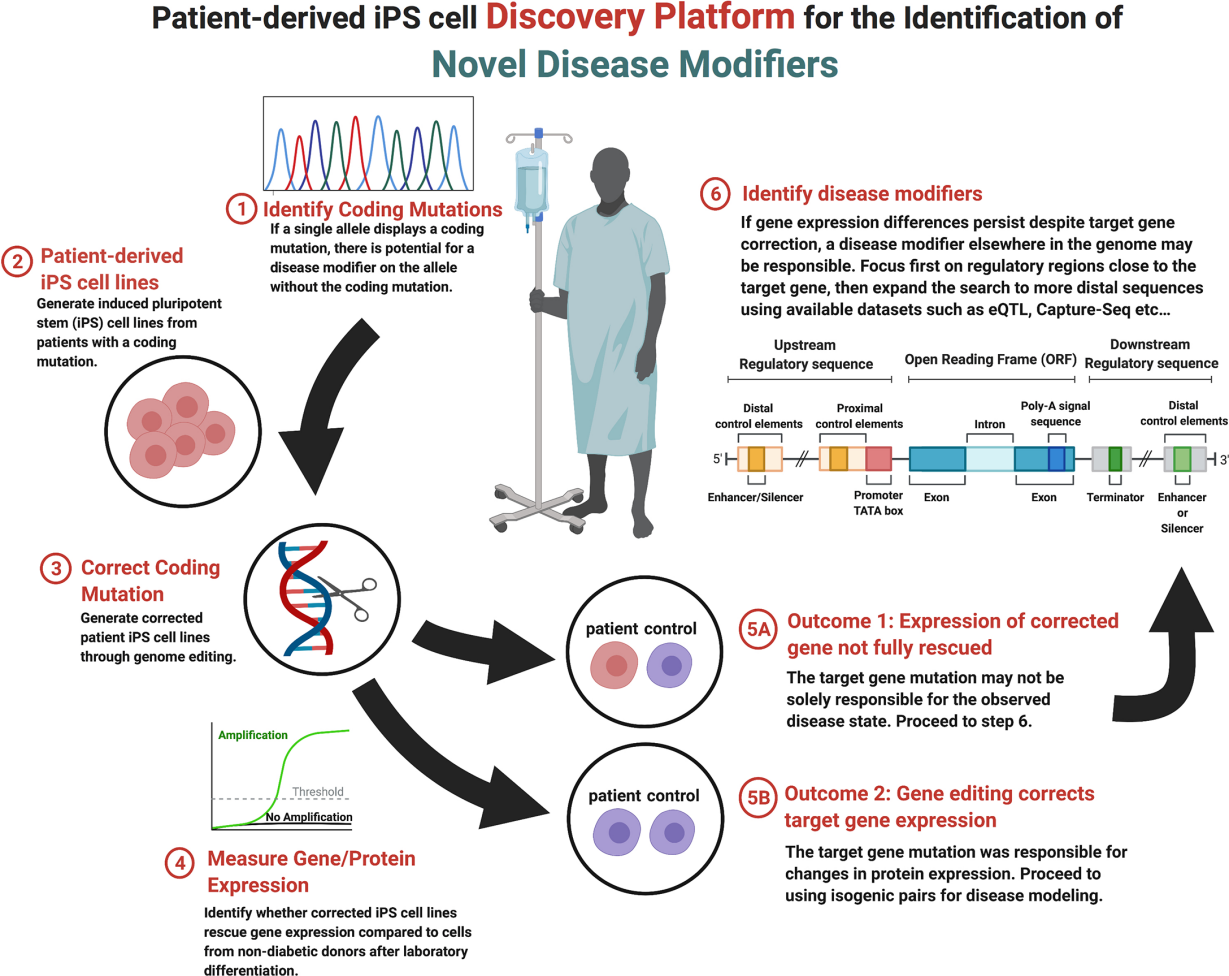
Stepwise flow diagram of the process of genetic disease modifier discovery.

The multifaceted nature of monogenic diabetes provides a unique opportunity to model gene-phenotype relationships that contribute to endophenotypes seen in the more common forms of the diabetes and aid in the discovery of novel disease modifiers (200, 201). Recent technological advancements in sequencing technology, genome editing (see *Section 2*), and the generation and guided-differentiation of iPSCs (see *Section 1.2*) now provide the foundation for an iPSC-based discovery platform that can identify novel protein coding and non-protein coding genetic elements that modify the presentation and penetrance of endophenotypes. Presented in **Figure 1**, genetic disease modifier discovery begins with the identification of a heterozygous coding mutation that results in monogenic diabetes. Coding variant identification can be done on demand through partial or whole genome sequencing given available information regarding candidate genes (97), through exome sequencing (98), or by leveraging publicly available GWAS datasets that compare non-diabetic and diabetic patients (99) (see *Section 3*). A useful database for monogenic diabetes includes the products of the DIAbetes Genetics Replication And Meta-analysis (DIAGRAM) Consortium (https://www.diagram-consortium.org/).

Upon candidate allele or gene selection, the next step within the disease modifier discovery platform is the production of iPSC lines from diabetic patients with the desired coding mutation (step 2, **Figure 1**). Due to recent technological advancements, iPSCs can easily be generated from adult cells that are harvested from blood or skin tissue (112, 202, 203). Once generated, endodermal cells with the desired coding mutation can be produced from iPSCs through exposure to the inductive signals that drive *in vivo* development (48, 204). To this end, several stepwise protocols that move cells through the multiple stages of pancreatic development *in vitro* over a few weeks have been developed (52–54). This process represents a relatively efficient method for the generation of pancreatic β-cells using tissue from multiple donors that share the identified coding mutation but have varied genetic backgrounds that result in different genetic modifiers, a distinct advantage when addressing observed heterogeneity in phenotypic expression.

After the generation of stable iPSC lines, the next step within the disease modifier discovery platform is to selectively correct the identified coding mutation and compare the resulting gene mRNA and protein expression before and after correction (step 3, **Figure 1**). As described in section 2, there are a number of genome editing technologies available to accomplish line correction, although CRISPR/cas9-mediated systems are becoming the most frequency used within stem cell models (63, 64). The goal of model generation is to compare corrected and non-corrected lines to non-diabetic controls, to which the mRNA and protein expression of the corrected gene can be measured (step 4, **Figure 1**). Throughout this process, one of two outcomes may be observed. *Outcome 1: the correction of the observed coding mutation can result in the complete normalization of gene/protein expression*. This result implies that the coding mutation was completely responsible for the decrease in gene expression and/or function (step 5A, **Figure 1**). *Outcome 2: the expression of the monogenic diabetes gene is not rescued to the levels observed in non-diabetic control cells*. In this case, expression is possibly being regulated by a modifier elsewhere in the genome (step 5B, **Figure 1**). It is possible that certain coding mutations may disrupt protein function while not impacting mRNA or protein expression. Such a mutation can still be studied with this platform as regulatory region variants which decrease gene expression may still be detectable when comparing the patient iPSC cell line to control lines.

In the event that the candidate gene expression is not normalized by selective correction of the coding mutation, the target gene may be under the regulatory control of one or more disease modifiers (step 6, **Figure 1**). Disease modifiers can reside either proximally or distally with respect to the coding mutation, as well as either upstream or downstream from the affected gene, making their location difficult to determine. An effective search strategy can be to focus on proximal regulatory regions near the gene of interest first, although if this approach doesn’t prove fruitful then there are a number of computational approaches that are specifically designed for the identification of regulatory elements [reviewed in (205)]. Similarly, the sequencing and chromatin mapping efforts of the ENCODE (https://www.encodeproject.org/) (206), Epigenome Roadmap Consortia (https://egg2.wustl.edu/roadmap/web_portal/) (207), and Common Metabolic Diseases Knowledge Portal (https://hugeamp.org) have provided extensive annotation of coding and non-coding regions within the human genome, as well as the likelihood of variants to impact metabolic disease.

Through the use of public databases, it is now possible to determine likely regulatory regions of the target gene of interest that can then be interrogated by targeted sequencing of patient iPS cell lines that could not be completely rescued by correction of the coding mutation. If variants are discovered, they can be studied by genome editing in the context of coding mutations or in isolation to determine the impact on expression of the target gene. For example, this strategy has been successfully used to study pancreas agenesis caused by mutations in GATA6 within our laboratory (102). A non-coding SNP was discovered in a patient iPS cell line that regulated expression of GATA6 during pancreas development *in vitro* and when interrogated in a cohort of patients with the disorder was confirmed to be a disease modifier. This strategy is especially useful in studying variants that impact genetic disease caused by heterozygous coding mutations. Variants that may have only a small influence on gene expression and no impact on disease alone can synergize with heterozygous coding mutations to bring target gene expression below a critical threshold needed for function. This platform does have some limitations including the requirement to focus of monogenic heterozygous disorders and may not be scalable to examine large numbers of genes with current differentiation technologies. We suggest that this methodology could be applied secondarily to any heterozygous iPSC disease modeling project that entails the creation of isogenic corrected lines with minimal additional effort.

## Conclusion and Future Outlook

The recent development of techniques to differentiate hPSCs into pancreatic β-cells has opened up new pathways to study the pathogenesis of diabetes. These human-centric models, combined with rapidly advancing genome editing techniques, provide incredibly powerful and scalable platforms in which to study the contribution of genetic elements to β-cell function, while also addressing the limitation of mouse models. Furthermore, the use of hPSCs provide unique opportunities in which to accomplish the targeted study of β-cell dysfunction as well as provide a platform to discover protein coding and non-protein coding genetic modifiers. Given recent evidence that large numbers of disease-linked variants do not reside in coding regions and the presence of variants can be population-specific, iPSC platforms that use patient-derived tissue hold great promise for the discovery of novel genetic disease modifiers that may help to explain the variability seen across and within diabetes subtypes.

While hPSC-based platforms represent a great leap forward in our ability to study β-cell function, there are caveats to their use that must be taken into account. hPSC derived β-cell generation and culture is labor-intensive, requiring approximately 40 days of differentiation and maturation. Additionally, though they do have some degree of insulin secretion in response to glucose and other secretagogues, significant uncertainty regarding their functionality and maturity still exists (66, 208–211). Ideally, these protocols need to be optimized to support the efficiency and accuracy of discovery platforms utilizing stem cell-derived β-cells. However, further fine-tuning of the established differentiation protocols will drive us closer to an ideal *ex vivo* human model of pancreatic β-cells. In addition to improving β-cell function, protocols need to be improved so that they are more universally successful, as certain hPSC lines can more easily differentiated into β-cells than others using current protocols.

The development of more universally-applicable protocols is required as the use of patient iPSC lines expands. There has been a recent flurry of publications that promise improved protocols with better function and wider applicability, and advances will continue to build on those already made (57, 212). Finally, generation of islet cells in platforms combining different stem cell-derived cell types will allow for more complex modeling of the genetic and environmental factors driving all forms of diabetes. Improving our knowledge of pancreatic β-cells function and development in humans is essential for the development of treatments for the millions of people affected by diabetes.

## Author Contributions

MG and KL conducted the literature review, designed figures, and wrote the manuscript. MG, KL, and PG conceived of the topic and edited the text. All authors contributed to the article and approved the submitted version.

## Funding

Authors were supported by grants K12DK94723 (KL), R01DK118155 (PG), UG3DK122644 (PG), R01DK123162 (PG) and UM1DK126194 (PG).

## Conflict of Interest

The authors declare that the research was conducted in the absence of any commercial or financial relationships that could be construed as a potential conflict of interest.
